# Small airway dysfunction in idiopathic pulmonary fibrosis

**DOI:** 10.3389/fphar.2022.1025814

**Published:** 2022-10-11

**Authors:** Chengsheng Yin, Huikang Xie, Xian He, Yuan Zhang, Aihong Zhang, Huiping Li

**Affiliations:** ^1^ Department of Pulmonary and Critical Care Medicine, Yijishan Hospital, The first Affiliated Hospital of Wannan Medical College, Wuhu, Anhui, China; ^2^ Department of Respiratory Medicine, Shanghai Pulmonary Hospital, Tongji University, School of Medicine, Shanghai, China; ^3^ Department of Pathology, Shanghai Pulmonary Hospital, Tongji University, School of Medicine, Shanghai, China; ^4^ Department of Medical Statistics, Tongji University, School of Medicine, Shanghai, China

**Keywords:** idiopathic pulmonary fibrosis, small airway dysfunction, mortality risk, combined with pulmonary fibrosis and emphysema, IPF combined obstructive ventilator dysfunction

## Abstract

It is generally accepted that the pathophysiology of idiopathic pulmonary fibrosis (IPF) can be attributed to impaired lung interstitium and alveoli, while airway involvement has rarely been reported. In the present study, we aimed to investigate the actual occurrence of IPF comorbid small airway dysfunction (SAD) and its impact on survival. Data from inpatients diagnosed with IPF at Shanghai Pulmonary Hospital (Shanghai, China) from 2011 to 2021 were retrospectively collected and analyzed. Lung function parameters were used to assess SAD. A total of 243 IPF patients were included in this retrospective study, and 84 cases (84/243, 34.57%) were diagnosed with SAD. The lung histopathology showed that all 48 cases undergoing lung transplantation presented various degrees of airway lesions, of which 18 patients (18/48, 37.5%) diagnosed with SAD before lung transplantation had a higher proportion of airway distortion and obliteration. The possible risk factors associated with IPF comorbid SAD were smoking, male, younger age, and high CT fibrosis and emphysema scores. By univariate Fine-Grey regression, the hazard ratio (HR) of IPF comorbid SAD was 1.725 (95% CI 1.071, 2.777, *p* < 0.05). After adjusting the CTPF model and GAP model, the value of HR was 1.714 (95% CI 1.043, 2.816, *p* < 0.05) and 1.731 (95% CI 1.074, 2.788, *p* < 0.05), respectively. These findings suggested that IPF comorbid SAD was an independent risk factor for the mortality of IPF patients.

## Introduction

Idiopathic pulmonary fibrosis (IPF) is the most common interstitial lung disease (ILD) (Raghu et al., 2018). Its pathophysiological process is generally recognized as that pulmonary interstitial hyperplasia leads to alveolar occlusion and damage. At the same time, the airways are spared, and the pulmonary function test (PFT) shows restrictive ventilation disorders, decreased total lung capacity (TLC), increased forced expiratory volume in one second (FEV1), normal or increased FEV1/FVC%, and decreased diffusing capacity for carbon monoxide (DLCO) (Gibson and Pride, 1977; Nicholson et al., 2002; Katzenstein et al., 2008). In recent years, IPF combined with pulmonary fibrosis and emphysema (CPFE) has been found in some studies, which usually exhibits fibrosis in the lower lobes combined with emphysema in the upper lobes (Cottin et al., 2005; Silva et al., 2008; Mitchell et al., 2015). Therefore, it is interesting to understand whether ILD/IPF patients combined with emphysema and bullae can cause airway dysfunction, which is a problem that has attracted the attention of scholars (Cottin et al., 2005). In 1977, Fulmer et al. (1977) have reported 12 patients (12/18, 66%) with airway stenosis in the lumen diameter of less than 2-mm air tube, and this stenosis lesion may be related to the patient’s clinical symptoms and disease severity by observing the pathological sections of 18 IPF patients by open-chest lung biopsy. Verleden et al. (2020) have compared pathological lung tissues of end-stage IPF patients undergoing lung transplantation with healthy donors without lung disease and found that the loss of terminal bronchioles is significant in IPF patients’ lung tissues. Guiot et al. (2022) have recently reported that about 15% of ILD patients have combined obstructive ventilator dysfunction diagnosed based on the PFT criteria FEV1/FVC < 70%, 7% (5/68) of IPF patients have obstructive ventilator dysfunction (O-IPF), and their small airway function is impaired significantly. Based on the above studies, we hypothesized that small airway dysfunction (SAD) might be a risk factor affecting the prognosis of IPF patients.

A review of the IPF genetics by Evans et al. (2016) has concluded that IPF is a complex genetic disorder with abnormal mucus cilia function, and the MUC5B promoter mutation rs35705950 is the most common risk factor for the development of IPF. Moreover, this mutation leads to overproduction of the mucus protein MUC5B, which obstructs the peripheral airways, leading to distortion, suppuration, and occlusion of small airways and resulting in pathological changes, such as infection and inflammatory cell infiltration. Some studies (Kirkham et al., 2008; Seibold et al., 2011) have reported that MUC5B is one of the primary mucins in the sputum of patients with chronic obstructive pulmonary disease (COPD). It can be seen that mutations in the MUC5B gene affect both IPF and COPD, which may be one of the mechanisms that complicate emphysema in IPF patients. The small airway is defined as bronchi with a lumen diameter of less than 2 mm (Macklem, 1998). SAD is commonly seen in COPD, bronchial asthma, and other chronic airway diseases (Halpin et al., 2021; Reddel et al., 2022). However, the incidence rate, major risk factors, and prognosis of IPF comorbid SAD remain unclear. At present, only very few clinical studies have focused on IPF comorbid SAD. Especially, there is a lack of large cohort studies to detect the incidence rate of IPF comorbid SAD and the impact on disease mortality.

In the present study, we explored the actual incidence rate of IPF comorbid SAD and its effect on mortality by retrospectively analyzing the lung function parameters, lung tissue pathology, and other clinical data of the IPF cohort. Collectively, our findings might help explore suitable preventive and therapeutic methods for them.

## Materials and methods

### Patient’s clinical data

**FIGURE 1:**
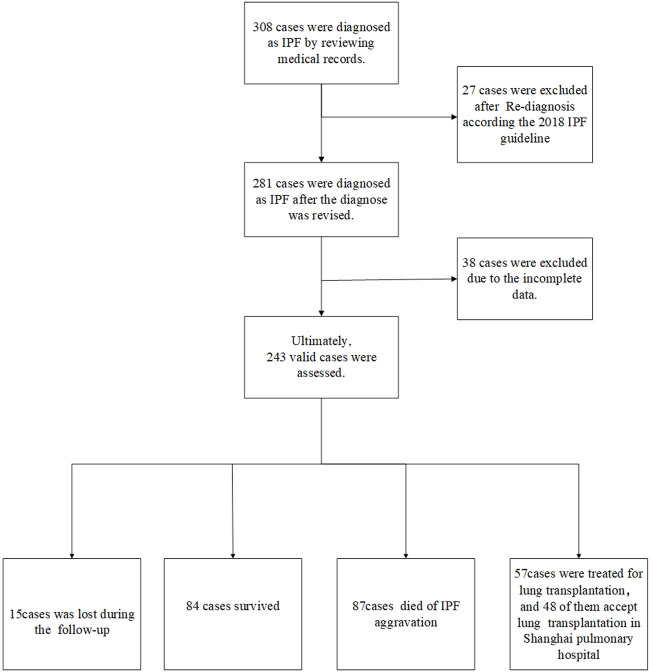
Case screening process A total of 308 cases were diagnosed with IPF by reviewing the medical records. Moreover, 27 cases were excluded after re-diagnosis according to the 2018 IPF guideline. Besides, 38 cases had incomplete CT and lung function data. Finally, 243 cases were included in the final retrospective analysis, including 57 cases undergoing lung transplantation. There were 84 surviving cases, and 15 cases were lost during follow-up. Besides, 87 deaths happened due to acute exacerbation of IPF, 57 patients underwent lung transplantation, and 48 of them accepted transplantation in Shanghai Pulmonary Hospital.

In the present study, the medical records and survival statuses of 308 patients who were diagnosed with IPF in the Department of Respiratory Medicine at Shanghai Pulmonary Hospital from 2011 to 2021 were retrospectively analyzed. The cases were reviewed according to diagnostic criteria of the 2018 IPF International Guidelines (Raghu et al., 2018) by a multidisciplinary team composed of two respiratory physicians, two radiologists, two thoracic surgeons, and two pathologists. Information, such as the patient’s gender, age, PFT paraments, SpO_2_% (or SaO_2_%), chest HRCT, occupation, and smoking history, was collected. The artificial intelligence HRCT pulmonary fibrosis assessment system developed by our team was adopted to calculate the extent of fibrosis and emphysema patterns (Wu et al., 2022). All patients were followed up by clinical visits and telephone follow-ups. The follow-up data included patient survival, time of death (the year and month of death), cause of death, other complications, whether undergoing lung transplantation, and the time of lung transplantation. In addition, two pathologists interpreted the lung histopathology of patients undergoing lung transplantation at Shanghai Pulmonary Hospital. All donor’s lungs were deemed appropriate for transplantation on review of the clinical files, and each patient undergoing transplantation signed informed consent. All data used in this study were approved by the Ethics Committee of Shanghai Pulmonary Hospital (No. K17-016). The flowchart of patient screening and enrollment and the follow-up results are presented in Figure 1.

### Small airway dysfunction diagnostic criteria by pulmonary function test

According to previous studies on lung function in the Chinese population (McFadden and Linden, 1972; Ciprandi et al., 2006; Perez et al., 2013; Lin et al., 2014; Xiao et al., 2020), three indicators of lung function were used to assess SAD, namely mid-maximum expiratory flow (MMEF), forced expiratory flow (FEF) at 50% of vital capacity (FEF50%), and FEF at 75% of vital capacity (FEF75%), and SAD was diagnosed when at least two of these three indicators were less than 65% of those predicted in the absence of bronchodilators.

### Histopathological diagnostic criteria for small airway dysfunction

According to the literature on small airway disease (Kawabata et al., 2008; Katzenstein et al., 2010; Chen et al., 2013; Koo et al., 2018; Higham et al., 2019), small airway lesions are often caused by inflammation or inflammatory injury response around the airway. In our present study, the pathologists evaluated the pathologic morphology of the small airway as follows. 1. Airway distortion: the wall thickness of the small airway was significantly increased (but the lumen was unobstructed), and the structure was distorted. 2. Airway obliteration: submucosal fibrous hyperplasia accompanied by significant stenosis or even complete occlusion of the small airway lumen. 3. Airway metaplasia: the alveolar septum around the bronchioles had slight fibrosis, and the alveolar epithelium around the bronchioles was replaced by cuboidal or columnar cells. 4. Airway inflammation and mucous: the number of inflammatory cells in small airway submucosa was significantly increased and caused embolization of the lumen.

### Statistical analysis

All analyses were performed using the statistical software IBM SPSS24.0, Stata/MP14.0, and GraphPad Prism 9. Measurement data were expressed as mean ± standard deviation (SD). Count data were presented as percentage (%) or proportion (%). The Kolmogorov-Smirnov test was used to analyze the distribution of variables. The *t*-test was used to analyze normally distributed variables in the bivariate analysis, while the Mann-Whitney *U*-test was used to analyze non-normally distributed variables. The chi-square test was used to compare qualitative variables. According to the collected data, logistic regression was used to determine the possible risk factors of IPF comorbid SAD. The variables that presented statistically significant differences (*p* < 0.05) in the bivariate analysis and were of clinical interest were included as the independent variables in the model. The forward stepwise technique (i.e., the Wald test) was then used to remove any variables with *p* > 0.1 from the final model. Patients’ survival time was defined from the date when patients’ data were acquired to the date of death endpoint or the last follow-up. The endpoint of this study was defined as the death caused by lung diseases. Lung transplantation was considered a competing risk event in this study. Other outcomes were treated as censored data. The Fine-Grey mortality risk regression model was adopted to analyze univariate risk and multivariate risk of SAD on the survival of IPF patients, and the hazard ratio (HR) and 95% confidence interval of each independent variable were calculated. A *p* < 0.05 was considered statistically significant.

## Results

### Patients’ clinical characteristics

**FIGURE 2:**
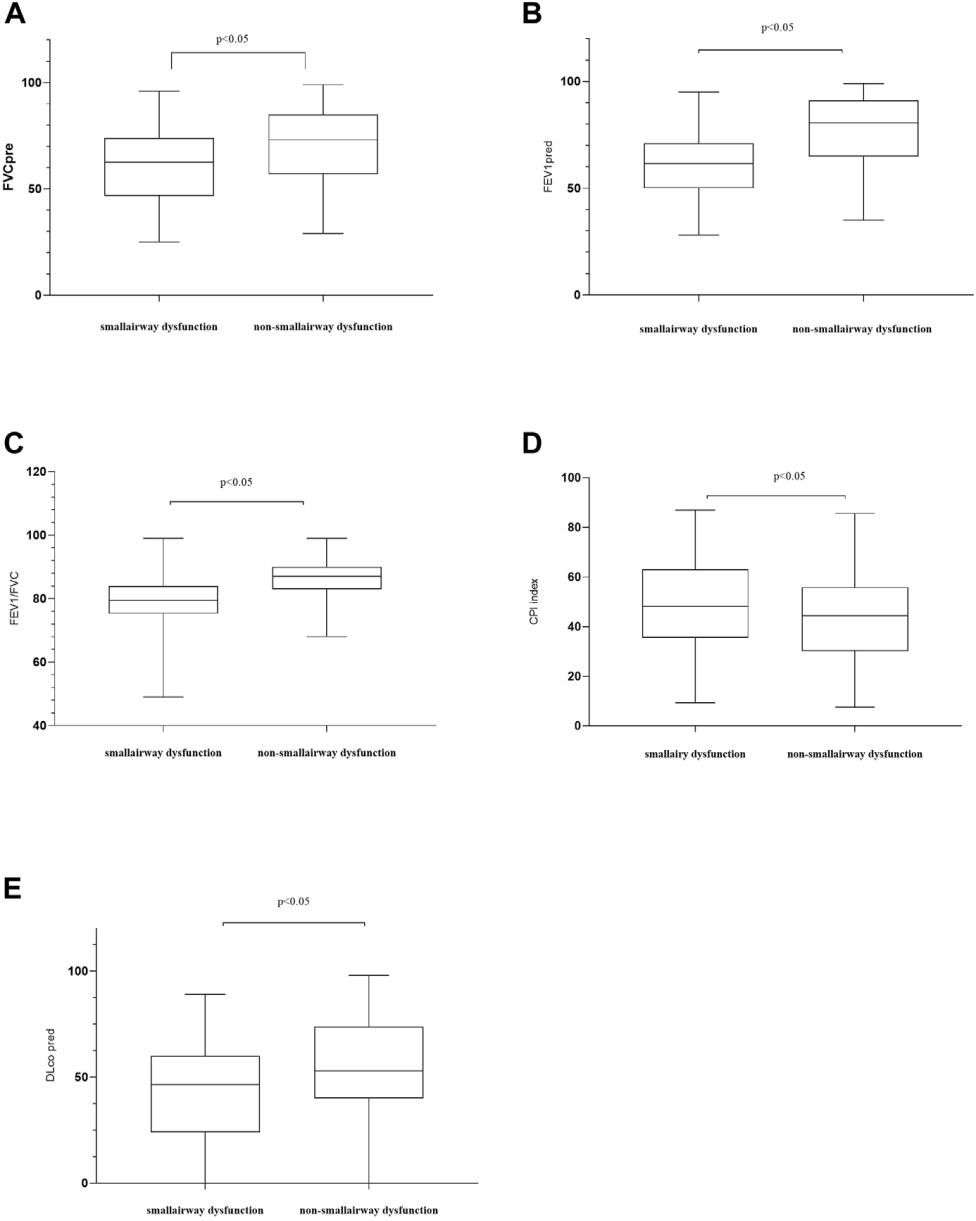
Comparisons of patients’ characteristics between the SAD and non-SAD groups. **(A)** FVC, forced vital capacity. FVC pred: the percentage of the actual FVC over the predicted FVC; **(B)** forced expiratory volume in 1 s (FEV1) % predicted; **(C)** FEV1/FVC ratio; **(D)** CPI, composite physiologic index. In 2002, Athol U. Wells and others proposed to use CPI, which combined chest CT and pulmonary functional parameters, to assess the severity of ILDs. A higher CPI represents a more severe ILD; **(E)** DLco, diffusing lung capacity for carbon monoxide. DLco% pred, the percentage of the actual DLco over the predicted DLco; Moreover, *p* < 0.05 indicates statistical significance.

Figure 1 illustrates the patient screening flow chart. A total of 308 IPF cases were included by reviewing the medical records. Moreover, 27 (27/308) cases were excluded according to the updated IPF guideline in 2018 (Raghu et al., 2018). Besides, 38 cases were excluded because of their incomplete data in CT and/or lung function. Finally, 243 cases were included in the final retrospective analysis. Of 243 IPF patients, 57 patients received lung transplants (48 of 57 performed in Shanghai Pulmonary Hospital). Other patients were treated by routine therapies, including oxygen therapy, antifibrotic drugs, and antioxidants. According to the diagnostic criteria of PFT, 84 patients (84/243, 34.57%) were diagnosed with IPF comorbid SAD, and 159 patients were non-SAD. Table 1 shows the case characteristics of the two groups. There was a statistical difference in lung function parameters between the two groups, while other clinical features had no significant difference (Figure 2). In addition, as shown in Table 2, 11 patients (11/84, 13%) in the SAD group were diagnosed with O-IPF by the PFT criteria FEV1/FVC < 70%. Compared with non-O-IPF patients, O-IPF patients had worse small airway lesions (FEF50 pred%, FEF75 pred%, and MMEF pred%) (*p* < 0.05). Moreover, O-IPF patients had lower FEV1 pred%, higher emphysema score, and lower median survival. However, the difference was not significant, which might be attributed to the small number of cases.

**TABLE 1:** Comparison of case characteristics.

	Small airway dysfunction	Non-small airway dysfunction	*P*-value
Case	84	159	
States (survive/death/lung transplantation)	25/38/21	74/49/36	0.028
Survive	25	74	0.013
Death	38	49	0.035
Lung transplantation	21	36	0.751
Male/female	74/10	149/10	0.130
Age	62.75 ± 8.50	64.7 ± 7.46	0.164
Smoking/Non-smoking	56/28	118/41	0.215
Fibrosis rate%	15.76 ± 11.16	14.24 ± 10.84	0.291
Emphysema rate%	1.59 ± 3.15	0.98 ± 1.80	0.117
Median survival time (months)	34 ± 7.59	52 ± 5.34	0.028
SpO_2_%	94.27 ± 4.14	94.94 ± 4.78	0.052
FVC	2.17 ± 0.73	2.50 ± 0.72	0.002
FVC pred%	62.86 ± 19.32	74.60 ± 21.01	<0.001
FEV1	1.68 ± 0.50	2.15 ± 0.55	<0.001
FEV1 pred%	60.89 ± 16.94	80.64 ± 21.01	<0.001
FEV1/FVC	79.55 ± 9.82	86.90 ± 5.57	<0.001
FEF25 pred%	67.00 ± 24.24	92.91 ± 23.72	<0.001
FEF50 pred%	62.49 ± 22.79	114.83 ± 30.98	<0.001
FEF75 pred%	39.9 ± 16.07	97.70 ± 44.22	<0.001
MMEF pred%	44.84 ± 14.38	94.24 ± 25.61	<0.001
DLcopred%	42.85 ± 25.00	55.44 ± 29.33	0.003
CPI index	50.54 ± 20.05	43.19 ± 20.47	0.014
CT stage((I/II/III)	13/54/17	36/99/24	0.317
PF grade(a/b/c)	26/40/18	74/62/23	0.055
GAP stage	31/36/17	88/48/23	0.010

Notes: Measurement data are presented as mean ± standard deviation (SD). Count data are presented as percentage or proportion. SpO_2_%, oxygen saturation of peripheral blood. SpO_2_ is the resting arterial oxygen saturation measured at fingertips. FVC, forced vital capacity; FVC% pred, the percentage of the actual FVC over the predicted FVC; FEV1, forced expiratory volume in one second. FEV1% pred, the percentage of the actual FEV1 over the predicted FEV1; DLco, diffusing capacity of the lung for carbon monoxide; DLco% pred, the percentage of the actual DLco over the predicted DLco; FEV1/FVC%, the percentage of FEV1 over FVC. Fibrosis Rate and Emphysema Rate calculated by AI according to the system established by Wu et al. (2022) FEF25 pred%, Forced expiratory flow at 25 of vital capacity; FEF50 pred%, Forced expiratory flow at 25 of vital capacity; FEF75 pred%, Forced expiratory flow at 75 of vital capacity; MMEF pred%, Maximal mid-expiratory flow; CT stage, The stage was determined by using AI according to the system established by Li HP et al. PF-based grade: The grade was determined by using the pulmonary function and physiological parameters (age, gender, FVC% pred, DLco% pred, and SpO_2_%) and following the CTPF model recommendation by Li HP et al. The grade was defined as: mild (a), moderate (b), and severe (c). GAP (gender, age, and physiologic variables) stage followed the recommendation by Brett Ley, and a higher stage represented a greater death risk. CPI: composite physiologic index. In 2002, Athol U. Wells and others proposed to use CPI, which combined chest CT and pulmonary functional parameters, to assess the severity of interstitial lung diseases (ILDs). A higher CPI represents a more severe ILD.

**TABLE 2:** Comparison between with and without obstructive ventilatory dysfunction in patients with IPF combined small airway dysfunction.

	O-IPF (FEV1/FVC <70%)	Non OIPF (FEV1/FVC >70%)	*P*
Cases	11	73	
FEF25 pred%	46.10 ± 24.12	70.14 ± 22.79	0.002
FEF50 pred%	29.56 ± 15.38	67.42 ± 19.37	<0.001
FEF75 pred%	22.65 ± 11.99	42.49 ± 15.02	<0.001
MMEF pred%	25.64 ± 14.55	47.73 ± 12.02	<0.001
Emphysema rate%	2.38 ± 2.37	1.48 ± 3.26	0.380
Fibrosis rate%	13.30 ± 9.40	16.13 ± 11.40	0.437
Median survival time (months)	20	27	0.237
SpO_2_%	93.18 ± 3.57	94.43 ± 4.22	0.352
FVC	2.51 ± 0.77	2.12 ± 0.71	0.096
FVC pred%	73.10 ± 18.25	61.31 ± 19.12	0.052
FEV1	1.57 ± 0.45	1.70 ± 0.51	0.462
FEV1 pred%	56.75 ± 16.72	61.52 ± 17.00	0.387
DLcopred%	52.94 ± 28.56	41.33 ± 24.23	0.152
CPI index	37.14 ± 20.52	52.56 ± 19.32	0.016

^a^ Airway obstruction was defined by a Tifeneau index FEV1/FVC <70%. Notes: Measurement data are presented as mean ± standard deviation (SD). O-IPF, combined obstructive and idiopathic pulmonary fibrosis. SpO_2_%, oxygen saturation of peripheral blood. SpO_2_ is the resting arterial oxygen saturation measured at fingertips. FVC, forced vital capacity; FVC% pred, the percentage of the actual FVC over the predicted FVC; FEV1, forced expiratory volume in one second; FEV1% pred, the percentage of the actual FEV1 over the predicted FEV1; DLco, diffusing capacity of the lung for carbon monoxide; DLco% pred, the percentage of the actual DLco over the predicted DLco. Fibrosis Rate and Emphysema Rate calculated by AI according to the system established by Li HP et al. FEF25 pred%, Forced expiratory flow at 25 of vital capacity; FEF50 pred%, Forced expiratory flow at 25 of vital capacity; FEF75 pred%, Forced expiratory flow at 75 of vital capacity; MMEF pred%, Maximal mid-expiratory flow; CPI, composite physiologic index. In 2002, Athol U. Wells and others proposed to use CPI, which combined chest CT and pulmonary functional parameters, to assess the severity of interstitial lung diseases (ILDs). A higher CPI represents a more severe ILD.

### Characteristics of patients receiving lung transplantation

**FIGURE 3:**
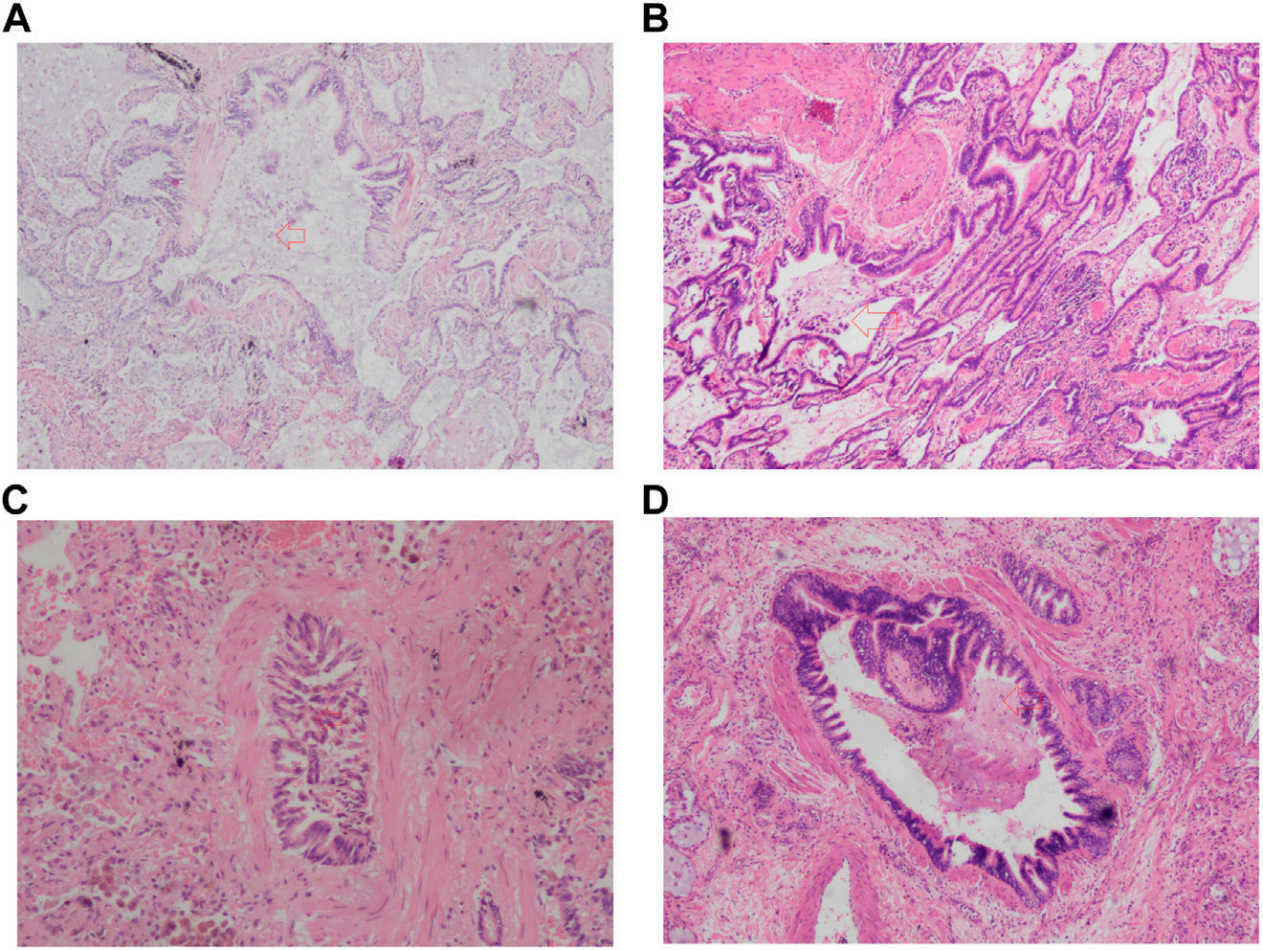
The pathologic image of SAD. **(A)** Airway distortion; **(B)** Airway metaplasia; **(C)** Airway obliteration; **(D)** Airway inflammation and mucous. The red arrow points to the lesion.

**FIGURE 4:**
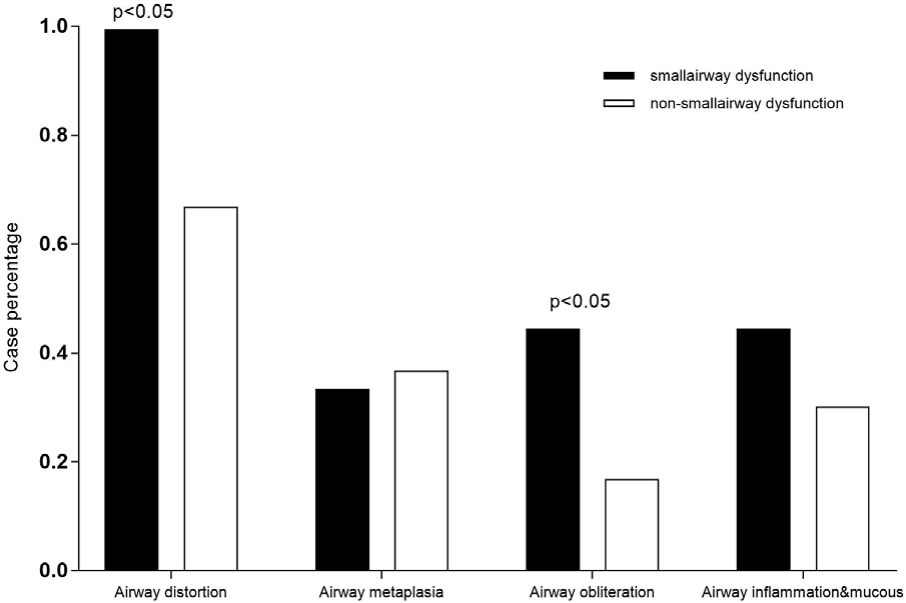
Comparisons of pathological characteristics between the SAD and non-SAD groups The pathological lesion types of airway distortion proportion and airway obliteration proportion were significantly high in SAD patients.

A total of 57 patients underwent lung transplantation. Among them, 48 patients accepted the operation at Shanghai Pulmonary Hospital, and their pathological lung tissue sections were preserved after transplantation. According to the latest PFT before transplantation, 18 patients (18/48, 37.5%) were diagnosed with IPF comorbid SAD. Table 3 lists the patient characteristics. The FEV1 pred% of the SAD group was lower (*p* = 0.004), and other lung function parameters showed no statistical differences. Patients receiving lung transplantation were younger in the SAD group (58, 66, *p* = 0.023). According to the assessment report of the pathological lung tissue section evaluated by the pathologist, all 48 cases exhibited pathological manifestations of usual interstitial pneumonitis (UIP) and had different degrees of airway lesions. Figure 3 shows the microscopic manifestations of pathological sections. Notably, Figure 4 and Table 3 reveal that the proportion of airway distortion and obliteration was higher in the SAD group.

**TABLE 3:** Characteristics of lung transplantation cases.

	Small airway dysfunction	Non-small airway dysfunction	*P*-value
Case	18	30	
Male/Female	13/5	27/3	0.110
Operation type			
BLTx/LSLTx/RSLTx	2/1/15	5/6/19	
Operation age	58 ± 11	66 ± 6.4	0.023
Smoking/Non-smoking	14/16	11/7	0.083
SpO_2_%	91.6 ± 9.4	91.2 ± 10.9	0.30
FVC pred%	55.0 ± 22.5	67.1 ± 24.5	0.083
FEV1 pred%	53.2 ± 19.8	74.0 ± 24.9	0.004
FVE1/FVC%	84.2 ± 12.9	89.0 ± 5.6	0.092
DLco pred%	37.8 ± 31.1	47.6 ± 23.9	0.327
Pathological interpretation (n/%)			
Airway distortion	17/94.4%	20/66.7%	0.027*
Airway metaplasia	6/33.3%	11/36.7%	0.815*
Airway obliteration	8/44.4%	5/16.7%	0.036*
Airway inflammationand mucous	8/44.4%	9/30.0%	0.311*

Notes: Measurement data are presented as mean ± standard deviation (SD). Count data are presented as percentage or proportion. BLTx, Bilateral lung transplant; LSLx, left-side lung transplant; RSLTx, right-sided lung transplant; SpO_2_%, oxygen saturation of peripheral blood. SpO_2_ is the resting arterial oxygen saturation measured at fingertips. FVC, forced vital capacity; FVC% pred, the percentage of the actual FVC over the predicted FVC; FEV1, forced expiratory volume in one second; FEV1% pred, the percentage of the actual FEV1 over the predicted FEV1; DLco, diffusing capacity of the lung for carbon monoxide; DLco% pred, the percentage of the actual DLco over the predicted DLco; FEV1/FVC%, the percentage of FEV1 over FVC. * The comparison is using the percentage.

### Risk factors of idiopathic pulmonary fibrosis comorbid small airway dysfunction

Table 4 shows the results of univariate and multivariate logistic regression analyses. Male patients with young age and high scores of pulmonary fibrosis and emphysema were more likely to be diagnosed with IPF comorbid SAD. However, *p* > 0.05 was found in both analyses. Therefore, independent risk factors associated with IPF comorbid SAD were not identified.

**TABLE 4:** Factors associated with small airway Dysfunction logistic regression analysis.

	Odd ratios (OR)	*P*-value	95% CI	
			Lower	Upper
Univariate analysis				
Smoking	1.439	0.216	0.809	2.560
Gender	2.014	0.136	0.803	5.051
Age	0.969	0.068	0.937	1.002
Fibrosis rate	1.013	0.304	0.989	1.037
Emphysema rate	1.111	0.067	0.993	1.243
Multivariate analysis				
Smoking	1.260	0.494	0.650	2.441
Gender	1.988	0.202	0.691	5.721
Age	0.967	0.067	0.934	1.002
Fibrosis rate	1.004	0.786	0.978	1.030
Emphysema rate	1.113	0.071	0.991	1.250

Notes: CI, confidence interval. Fibrosis rate, the percentage of fibrosis in chest CT calculated by AI according to the system established by Li HP et al.; Emphysema rate, the percentage of Emphysema in chest CT calculated by AI according to the system established by Li HP et al.

### Survival analysis

**FIGURE 5:**
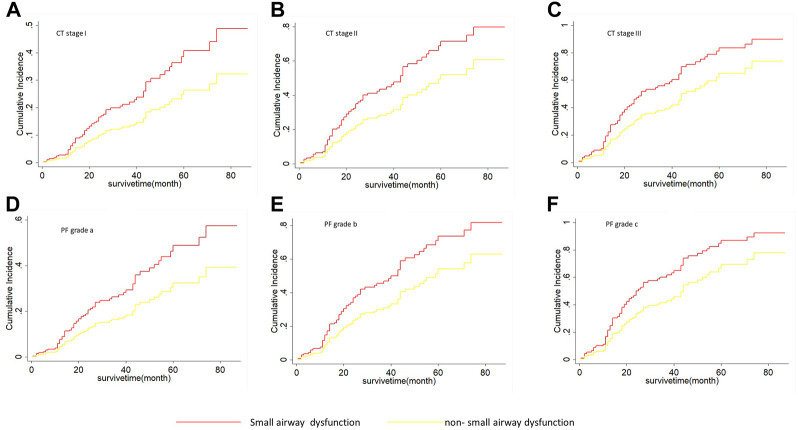
The cumulative risk of death with SAD and non-SAD in different CTPF stages based on the Fine-Gray regression model. **(A)** Cumulative death risk curve of patients with SAD and non-SAD in CT stage I; **(B)** Cumulative death risk curve of patients with SAD and non-SAD in CT stage II; **(C)** Cumulative death risk curve of patients with SAD and non-SAD in CT stage III; **(D)** Cumulative death risk curve of patients with SAD and non-SAD in grade a; **(E)** Cumulative death risk curve of patients with SAD and non-SAD in grade b; **(F)** Cumulative death risk curve of patients with SAD and non-SAD in grade c. As the cumulative dearth risk curve show, IPF comorbid SAD had a high dearth risk in all different CTPF stages. The red curve indicates that PFT diagnoses SAD. The yellow curve indicates that PFT diagnoses non-SAD. Note: CTPF model: A Mortality Risk Prediction model used to evaluate the severity of IPF based on Artificial Intelligence which was established by Li HP et al. CT stage: The stage was determined by using AI according to the system installed by Li HP et al. CT I: Honeycomb area rate was <5% of the entire lung. CT II: Honeycomb area rate was 5%–25% of the entire lung. CT III: Honeycomb area rate was >25%. PF grade: The grade was determined by using the pulmonary function and physiological parameters (age, gender, FVC% pred, DLco% pred, and SpO_2_%). The grade was defined as: mild **(A)**, moderate **(B)**, and severe **(C)**.

**FIGURE 6:**
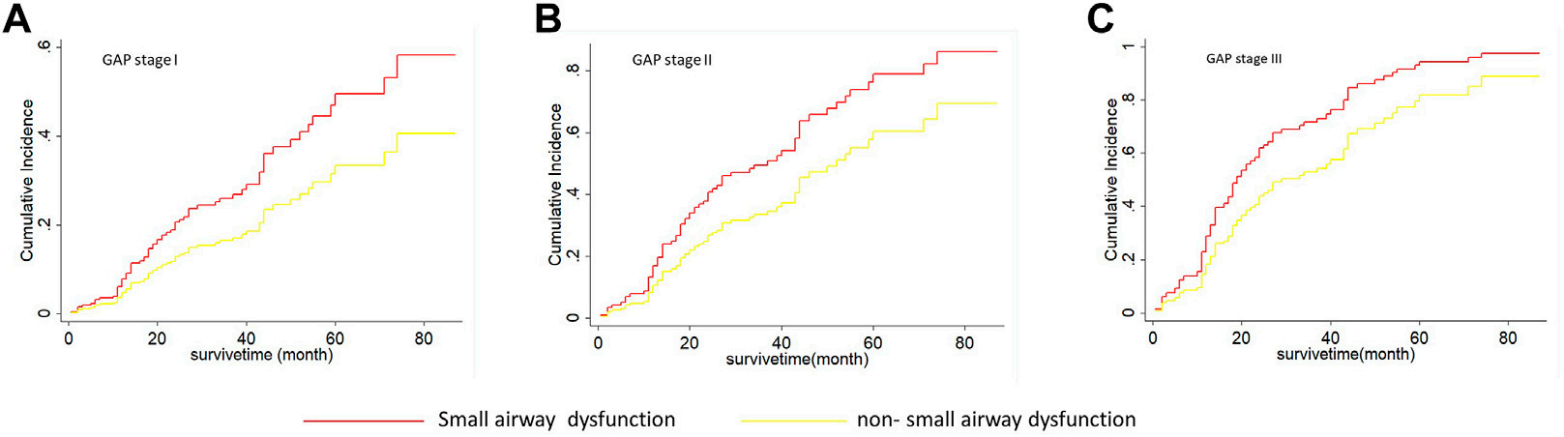
The cumulative risk of death with SAD and non-SAD in different GAP stages based on the Fine-Gray regression model. **(A)** Cumulative death risk curve of patients with SAD and non-SAD in GAP stage I; **(B)** Cumulative death risk curve of patients with SAD and non-SAD in GAP stage II; **(C)** Cumulative death risk curve of patients with SAD and non-SAD in GAP stage III; IPF comorbid SAD had the high dearth risk in all GAP stages. Note: The red curve indicates that PFT diagnoses SAD; the yellow curve indicates that PFT diagnoses non-SAD. GAP (gender, age, and physiologic variables) stage followed the recommendation by Brett Ley, and a higher stage represented a greater death risk.

As shown in Table 5, on univariate Fine-Grey competitive risk regression analysis of IPF comorbid SAD, the HR value was 1.725 (95% CI: 1.071, 2.777, *p* < 0.05). After adjusting the CT fibrosis stage and pulmonary function, as well as physiological condition grade (CTPF model) and GAP model, the HR value became 1.714 (95% CI: 1.043, 2.816, *p* < 0.05, *p* < 0.05) and 1.731 (95% CI 1.074, 2.78). According to mortality risk curves after adjusting the different assessment predict models shown in Figures 5, 6, SAD patients had a higher risk of death than non-SAD patients in all models. These results suggested that patients with IPF comorbid SAD could be an independent risk factor for mortality.

**TABLE 5:** Fine-gray death risk regression analysis results from different model factors.

	Hazard ratio (HR)	*P*-value	95% CI	
			Lower	Upper
Univariate factors				
Small airway dysfunction	1.725	0.025	1.071	2.778
Multivariate factors (Adjust CTPF model)				
Small airway dysfunction	1.714	0.033	1.043	2.816
CT I	referent			
CT II	2.393	0.017	1.171	4.894
CT III	3.425	0.009	1.368	8.571
PF(a)	referent			
PF(b)	2.000	0.014	1.151	3.465
PF(c)	3.021	0.004	1.420	6.424
Multivariate factors (Adjust GAP stage)				
Small airway dysfunction	1.675	0.035	1.038	2.705
GAP I	referent			
GAP II	2.276	0.002	1.356	3.820
GAP III	4.189	<0.001	2.134	8.222

Notes: CI, confidence interval; CTPF model, A Mortality Risk Prediction model used to evaluate the severity of IPF based on Artificial Intelligence which established by Li HP et al. CT stage: The stage was determined by using AI according to the system established by Li HP et al. CT I: Honeycomb lesion area was <5% of the entire lung. CT II: Honeycomb lesion area was 5%–25% of the entire lung. CT III: Honeycomb lesion area was >25%. PF grade: The grade was determined by using the pulmonary function and physiological parameters (age, gender, FVC% pred, DLco% pred, and SpO_2_%) and following the CTPF model recommendation by Li HP et al. The grade was defined as: mild (a), moderate (b), and severe (c). GAP (gender, age, and physiologic variables) stage followed the recommendation by Brett Ley, and a higher stage represented a greater death risk.

## Discussion

The pathophysiology of IPF is generally considered to be impaired lung interstitium and alveoli but rare airway involvement (Mead, 1970; Gibson and Pride, 1977; Nicholson et al., 2002). Our retrospective analysis consisting of 243 IPF patients showed that one-third (84/243, 34.57%) of IPF patients had SAD, and these patients had a significantly higher mortality risk compared with non-SAD patients (HR 1.725, *p* < 0.05), and the median survival was significantly shortened (34 ± 7.59 vs. 52 ± 5.34 months). Further stratified analysis showed that IPF comorbid SAD at different CTPF stages and GAP stages had a substantially higher risk of death compared with those non-SAD patients. Previous studies on IPF mortality prediction have mostly focused on FVC, DLCO, chest CT pulmonary fibrosis range, age, gender, and other parameters (King et al., 2001; Zappala et al., 2010; Ley et al., 2014), while no large-scale case-cohort studies have focused on SAD in IPF patients. In the present study, the sample size of IPF patients in our cohort reached 243, and our study showed that IPF comorbid SAD was an independent risk factor associated with the mortality of IPF patients.

Among the 84 patients diagnosed with SAD, only 11 cases (11/84, 13%) had significant obstructive ventilatory dysfunction (FEV1/FVC < 0.7), which was slightly higher than the value of 7% (5/68) reported by Guiot et al. (2022). In contrast, the majority of patients (87%) showed no significant obstructive ventilatory dysfunction by routine PFT. The results showed that the changes in small airway function indexes (MMEF, FEF50%, FEF75%) were more sensitive than normal airway function indexes (FEV1/FVC < 70%), by which abnormalities in IPF patients could be detected earlier. Table 2 shows that once IPF was combined with O-IPF, their SAD became worse (*p* < 0.001), and their median survival time was shortened.

The histopathology of patients undergoing lung transplantation in our cohort showed that all 48 IPF patients had diverse degrees of small airway lesions in their pathological section, while the proportion of airway distortion and obliteration was higher in patients with SAD diagnosed by the preoperative PFT (Table 3). The lesions of airway distortion and obliteration might be more critical pathological changes affecting SAD. SAD patients undergoing lung transplantation were younger compared with the non-SAD group, suggesting that patients with SAD progressed to the end stage of the disease more quickly. Some studies have found that in end-stage ILD, there is a significant reduction in the number of small terminal airways, showing loss of terminal bronchioles, fibroblastic foci, lymphocyte-dominated immune cell infiltration inflammation, increased lymphatic follicular volume fraction, and even emphysema with increased alveolar volume and more severe clinical symptoms through micro-CT, new *in-vivo* imaging methods, and other means (Fulmer et al., 1977; Kawabata et al., 2008; Katzenstein et al., 2010; Galban et al., 2012; Chen et al., 2013; Koo et al., 2018; Higham et al., 2019; Vasilescu et al., 2019; Verleden et al., 2020), which can partially explain why IPF patients are combined with small airway lesions and develop to CPFE eventually. Our study confirmed that the small airway lesion was a common feature of all end-stage IPF diseases. Based on our research, we speculated that the progression of IPF damaged pulmonary alveoli and interstitium first by fibroblast proliferation. Then it spread to the small airway to gradually impair the small airway function, finally invading the large airway. PFT would show apparent ventilation dysfunction when the airway obliteration and distortion developed severely. Therefore, clinicians should pay more attention to the changes in small airway function in IPF patients, adjust the therapeutic measures timely, and improve the effectiveness of treatment outcomes.

Previous studies of SAD have mainly focused on chronic airway diseases, such as COPD and asthma, and the risk factors associated with SAD are air pollutant exposure and smoking (Konstantinos Katsoulis et al., 2016; Usmani et al., 2016). Few clinical studies have focused on ILD with small airway lesions. Guiot et al. (2022) have reported that about 15% of ILD patients are complicated with obstructive ventilator dysfunction. Among them, 7% (5/68) of patients have IPF comorbid obstructive ventilator dysfunction and impaired small airway function. However, due to the small sample size, the risk factors are not analyzed. Our case-cohort logistic regression analysis found that patients with IPF comorbid SAD had higher proportions of smoking, male, younger age, and a larger range of pulmonary fibrosis and emphysema patterns in chest CT. However, the *p* values were not statistically significant in both multivariate and univariate regression analyses. Therefore, the independent risk factors associated with IPF comorbid SAD needed to be analyzed in a larger sample, multicenter, and long-term follow-up cohort.

There were several limitations in our study. First, even though our research found that O-IPF (11 cases) patients had a higher proportion of emphysema and shorter survival time, there was no significant difference due to the small sample size. Second, the detailed therapeutic process was not analyzed because of the retrospective analysis. Whether the patients with IPF comorbid SAD need to be treated by inhaled glucocorticoid and bronchodilators like COPD or other chronic airway diseases? Whether the patients can benefit from this treatment or not? All these questions need to be answered in future studies. Third, this study was a single-center retrospective analysis, and multicenter prospective studies with large samples are required.

In conclusion, we found that 30% of IPF patients had comorbid SAD, and the mortality risk of these patients was significantly higher compared with the non-SAD group. Moreover, no significant obstructive ventilatory dysfunction was found in 87% of patients with IPF comorbid SAD. When IPF patients had combined significant ventilatory dysfunction, the degree of SAD was worse, and the median survival time was shorter. These findings suggested that clinicians should pay more attention to the small airway function in IPF patients, and appropriate treatment should be explored.

## Data Availability

The raw data supporting the conclusion of this article will be made available by the authors, without undue reservation.

## Abbreviations

IPF, idiopathic pulmonary fibrosis; ILD, interstitial lung disease; PFT, pulmonary function test; TLC, total lung capacity; FVC, forced vital capacity; DLCO, diffusing capacity of the lung for carbon monoxide; CPFE, combined with pulmonary fibrosis and emphysema; O-IPF, IPF patients have obstructive ventilator dysfunction; FEV1, forced expiratory volume in one second; COPD, chronic obstructive pulmonary disease; SAD, small airway dysfunction; CT, computed tomography; CPI, composite physiologic index; GAP, gender, age, and physiologic variables; SaO_2_, oxyhemoglobin saturation; SpO_2_, oxygen saturation of peripheral blood; HRCT, high-resolution computed tomography; UIP, usual interstitial pneumonia; PF, pulmonary function and physiological features; CTPF, HRCT combined pulmonary function andphysiological features.
